# Hydrology influences breeding time in the white-throated dipper

**DOI:** 10.1186/s12898-020-00338-y

**Published:** 2020-12-17

**Authors:** Anna L. K. Nilsson, Thomas Skaugen, Trond Reitan, Jan Henning L’Abée-Lund, Marlène Gamelon, Kurt Jerstad, Ole Wiggo Røstad, Tore Slagsvold, Nils C. Stenseth, L. Asbjørn Vøllestad, Bjørn Walseng

**Affiliations:** 1 Centre for Ecological and Evolutionary Synthesis (CEES), Department of Biosciences, University of Oslo, P. O. Box 1066, Blindern, 0316 Oslo, Norway; 2 Norwegian Institute for Nature Research, Thormøhlens Gate 55, 5006 Bergen, Norway; 3 Norwegian Water Resource and Energy Directorate, P. O. Box 5091, Majorstua, 0301 Oslo, Norway; 4 Centre for Biodiversity Dynamics, Department of Biology, Norwegian University of Science and Technology, 7491 Trondheim, Norway; 5 Jerstad Viltforvaltning, Aurebekksveien 61, 4516 Mandal, Norway; 6 Faculty of Environmental Sciences and Natural Resource Management, Norwegian University of Life Sciences, P.O. Box 5003, NMBU, 1432 Ås, Norway; 7 Norwegian Institute for Nature Research, Gaustadallén 21, 0349 Oslo, Norway

**Keywords:** Breeding phenology, Climate change, Environmental heterogeneity, Hydropower, Long-term study, Passerine bird, River discharge, Snow, Spatial scale, Stream

## Abstract

**Background:**

Earlier breeding is one of the strongest responses to global change in birds and is a key factor determining reproductive success. In most studies of climate effects, the focus has been on large-scale environmental indices or temperature averaged over large geographical areas, neglecting that animals are affected by the local conditions in their home ranges. In riverine ecosystems, climate change is altering the flow regime, in addition to changes resulting from the increasing demand for renewable and clean hydropower. Together with increasing temperatures, this can lead to shifts in the time window available for successful breeding of birds associated with the riverine habitat. Here, we investigated specifically how the environmental conditions at the territory level influence timing of breeding in a passerine bird with an aquatic lifestyle, the white-throated dipper *Cinclus cinclus*. We relate daily river discharge and other important hydrological parameters, to a long-term dataset of breeding phenology (1978–2015) in a natural river system.

**Results:**

Dippers bred earlier when winter river discharge and groundwater levels in the weeks prior to breeding were high, and when there was little snow in the catchment area. Breeding was also earlier at lower altitudes, although the effect dramatically declined over the period. This suggests that territories at higher altitudes had more open water in winter later in the study period, which permitted early breeding also here. Unexpectedly, the largest effect inducing earlier breeding time was territory river discharge during the winter months and not immediately prior to breeding. The territory river discharge also increased during the study period.

**Conclusions:**

The observed earlier breeding can thus be interpreted as a response to climate change. Measuring environmental variation at the scale of the territory thus provides detailed information about the interactions between organisms and the abiotic environment.

## Background

Spring phenology, the annual timing of recurring life-history events in spring, is well-known to have advanced (become earlier) in response to climate change [42]. Earlier breeding in birds is one of the strongest responses to global change and is a key factor determining reproductive success [7, 37, 54]. Most studies of climatic effects have focused on large-scale estimates of environmental variation, such as the North Atlantic Oscillation (NAO) or temperature averaged over large geographical areas, neglecting the fact that organisms are affected by the local conditions in their home ranges [34]. Even on the scale of a population, an individual might use a very limited proportion of the population space; thus, population-specific measurements might not accurately reflect the exposure of an individual to the current environmental conditions [11, 24]. In fact, organisms experience the local weather and its direct effects on the availability of food and shelter. This determines whether resources are to be accumulated or if stored resources are depleted, and determines the allocation of resources to growth, survival and reproduction. In addition to the local weather conditions, the environmental challenges faced by organisms may also be influenced by other aspects of the local area they inhabit. The local area is often defended against conspecifics and is then termed a territory. Territories are heterogeneous in geography and topography, which can have implications for the availability of breeding sites and foraging opportunities; the availability of food resources might vary at small spatial scales [24]. The robustness to environmental variation such as droughts and floods might furthermore vary between territories. Such heterogeneity can lead to differences in territory quality [46] determining variation in occupancy between years [13]. Environmental variation measured at the scale of the territory might thus affect individual life history decisions, such as the timing of breeding.

Temperature and precipitation have increased rapidly during the last 100 years in northern Europe [22]. This has naturally led to changes in snow conditions and the extent of ice cover during winter [47] and potentially changed the timing of snow melt, and ultimately the timing of spring floods. Similarly, this has changed water discharge in rivers and streams, and consequently the occurrence and size of floods and droughts [29]. Further climate change is predicted to lead to more alterations in precipitation, discharge, evaporation, and shifts in drought and flood patterns [33], which will have important implications for species closely associated with the riverine habitat [43]. In addition, freshwater ecosystems are under pressure in the pursuit of clean and renewable energy resources, where hydropower is an attractive alternative to fossil fuels. Hydropower development has major impacts on ecosystem functioning as the construction of dams and reservoirs interrupts the natural flow regime, which is the main driver of all ecological processes in rivers and streams [39].

In birds, most studies of breeding phenology have focused on species that depend on terrestrial food resources [7, 14, 55], but see [10]. Here, we report the results from a long-term study of a passerine bird that collects most of its resources year-round under water, namely the white-throated dipper *Cinclus cinclus* (hereafter dipper). This aquatic life style confers some special challenges in the northern range of the dippers’ distributions, because winter survival and onset of breeding are dependent on the presence of ice-free (open) water [18, 35, 37], and thus on hydrological dynamics. Spring temperature is important for initiating breeding, but spring temperature has so far only been measured at the regional level [37]. The documented effect of spring temperature most likely reflects an extrapolation of local microclimatic conditions on the scale of the territory, such as local river discharge dynamics and snow conditions. Thus, in this study, we investigate how environmental variation at the scale of the territory, such as the local river discharge (defined as the volume of water flowing past a cross-section of a river per unit time), hydrological dynamics, territory and individual characteristics, influence the timing of breeding. In this study system, Skaugen et al. [48] developed a model allowing simulations of daily values of a number of biologically important hydrological variables at each dipper territory in our study system during 1978–2015. We here relate these unique hydrological data to our long-term dipper breeding data to test the hypothesis that local environmental variation, particularly runoff, at the scale of the territory influences timing of breeding. Furthermore, our study uses a novel method, the biologically-based “trigger date”, to deal with the statistically unsound problem of circularity in causality. This is often encountered in phenology analyses when using sliding window-approaches to determine periods of importance, which then are included in the statistical analyses (using response to find appropriate period and then using period to predict response).

## Results

### General analyses

**Fig. 1:**
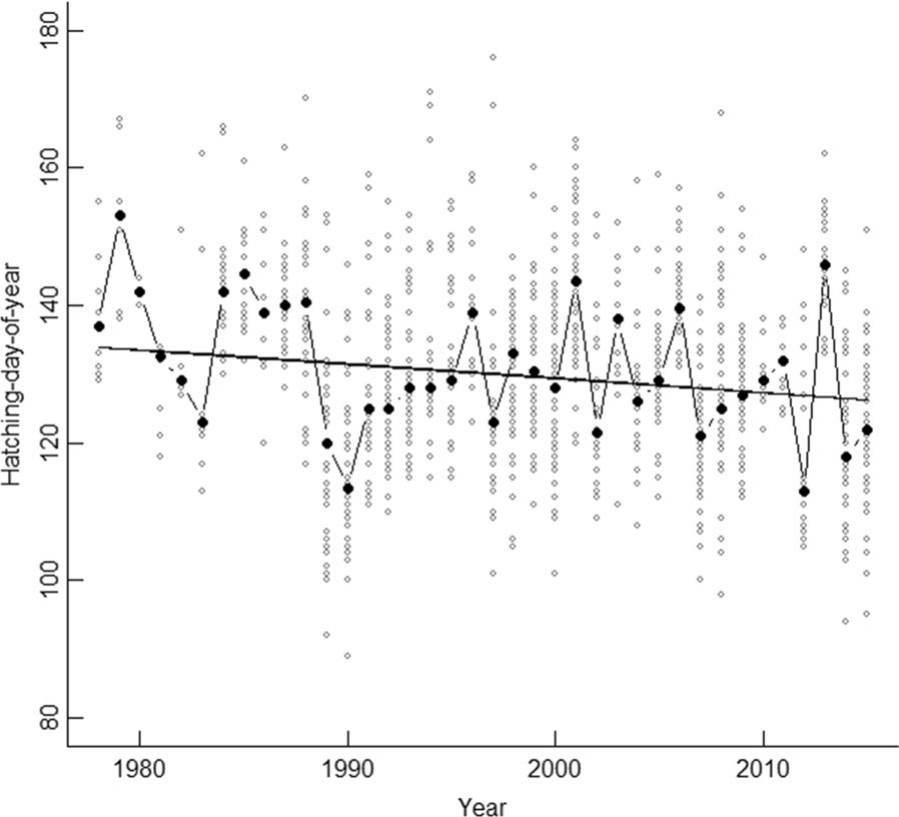
The advancement in breeding time in the white-throated dipper population in Lyngdalselva. The advancement in hatching date (hatching day-of-year, solid line) in the white-throated dipper population in Lyngdalselva, in Norway. Grey points denote observed annual hatching days and black points the observed annual average hatching date in 1978–2015

The median hatching date of the dipper was the 8th of May (hatching-day-of-year = 128, mean = 129.6, standard deviation, SD = 13.2 days). Hatching date advanced (earlier) 10.2 days during the study period (1978–2015) as shown in a mixed-effects linear regression model with only year as a linear predictor and year as the random effect (random effect, year SD = 7.7, residual SD = 10.9; fixed effect year: b = − 0.27, t = − 2.2, P = 0.035; Fig. 1). Male dippers arrived at the breeding grounds 2.5 days before females (mean = 2.43, SD = 18.65; t = 6.2, df = 2278, P < 0.0001). Trigger dates varied from 1st of February (day-of-year = 31) to 28th of April (day-of-year = 119; median = 43 (13th of Feb), mean = 54.9 (24th of Feb)), and the annual variation in trigger dates among territories was on average 38 days (SD = 25.3, range 0–87). In random factor decomposition, we found a standard deviation of 10.6 days between the territories, while the within-territory standard deviation was 23.0 days. This means that the within-territory variation explained 82.6% of the variation in trigger date. During the study period, there was a positive temporal trend in the relative winter discharge (trigger period 17 (Dec–Feb; see Table S1, Additional file 1); mixed-effects linear model: random effect: year SD = 0.53, residual SD = 0.28, fixed effect year: b = 0.02, t = 2.1, P = 0.04).

### Model selection

**Fig. 2:**
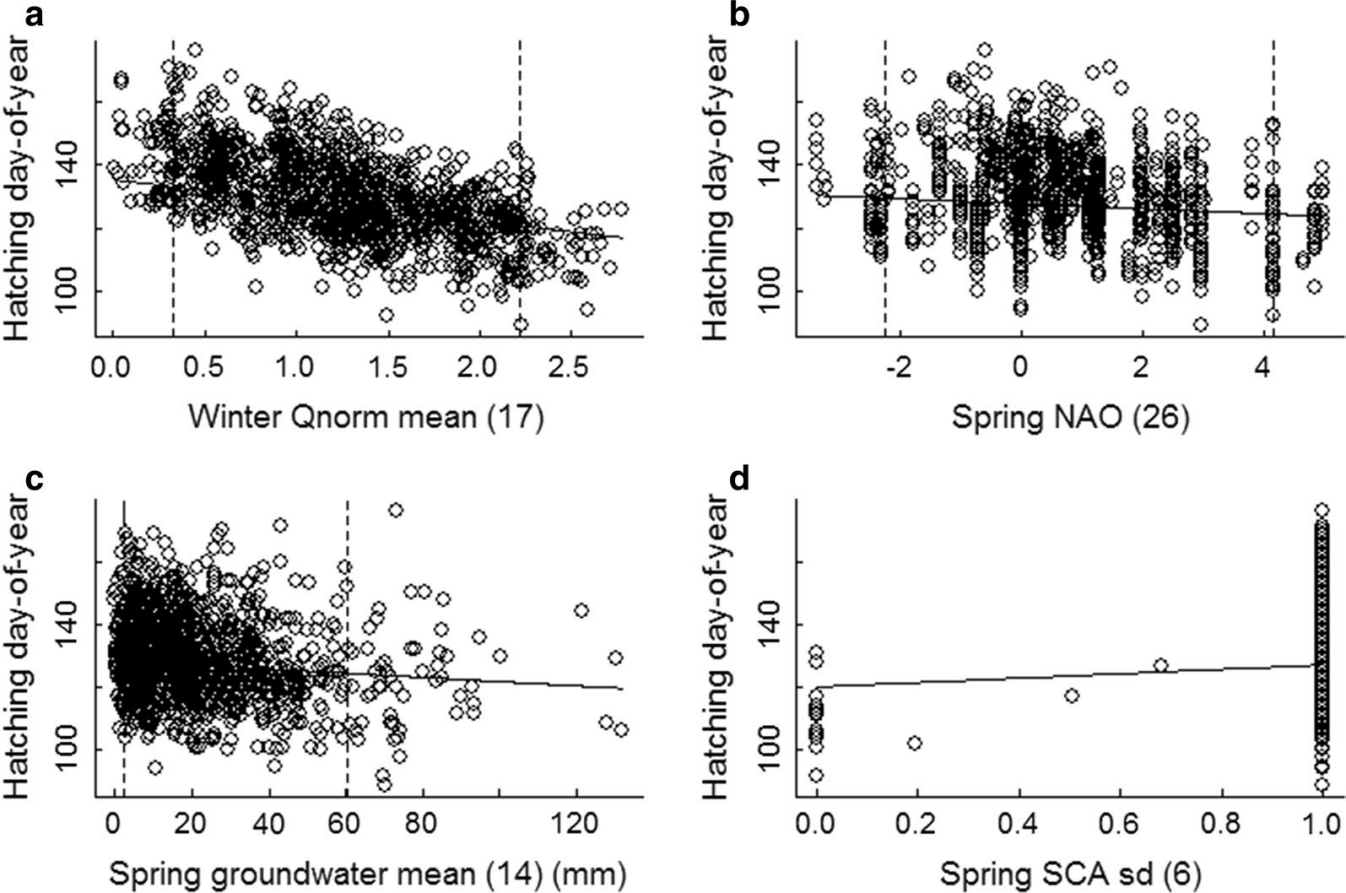
The influence of the hydrological and climatic predictor variables on the timing of breeding. The influence of the hydrological and climatic predictor variables on hatching date (hatching day-of-year) in the white-throated dipper population in Lyngdalselva 1978–2015. **a** Mean relative river discharge at each territory during the preceding Dec–Feb (Winter Qnorm mean [17]), **b** the North Atlantic Oscillation (NAO) 10–21 days after the trigger date at each territory (Spring NAO [26], **c** the minimum ground water levels for the last 20 days before the trigger date (Spring groundwater mean [14]), and **d** the maximum Snow Covered Area (SCA) the last 30 days before the trigger date (Spring SCA sd (06)), where 0 is no snow cover and 1 is full snow cover. Solid lines denote the effect and vertical dotted lines denote the 5 and 95% quantiles of the raw data. The trigger date is defined as the first date when the daily air temperature exceeded 0 °C for five consecutive days

**Fig. 3:**
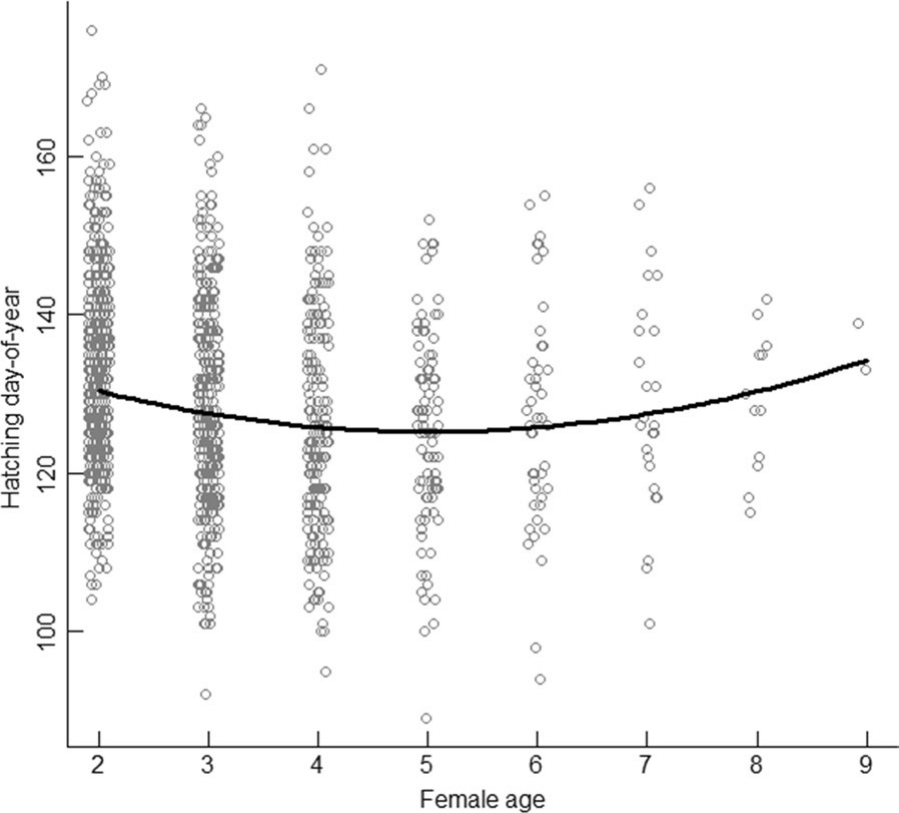
The effect of age on timing of breeding. The effect of age (calendar year) on timing of breeding (hatching day-of-year) shown as a quadratic line (for all other variables set at their average values) in the white-throated dipper population in Lyngdalselva 1978–2015. Grey dots correspond to observed hatching dates per age, with ages jittered in order to increase visibility of the full dataset

**Fig. 4:**
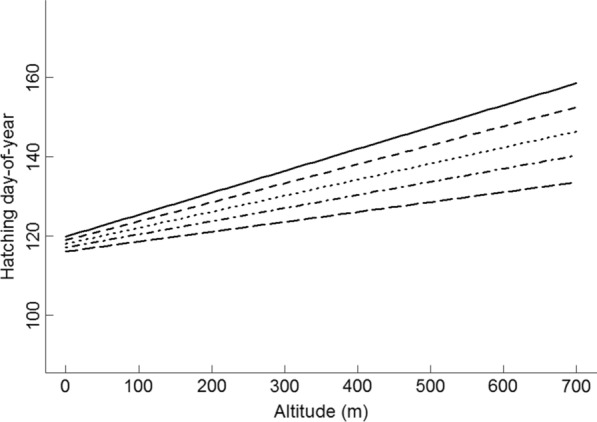
The effect of altitude on the timing of breeding. The effect of altitude (all other variables set at their average values) on the timing of breeding (hatching day-of-year) in 1978 (solid line), 1987 (stapled line), 1996 (dotted), 2005 (stapled and dotted), 2015 (long staples) in the white-throated dipper population in Lyngdalselva 1978–2015

The most supported model to account for the variation in hatching date in the dipper population contained the environmental variables normalized mean winter river discharge (Winter Qnorm mean 17), spring NAO (Spring NAO 26), spring ground water levels (Spring Groundwater mean 14), spring variation in snow cover (Spring SCA sd 6), and the temporal trend in altitude, where the effect of altitude on timing of breeding changed during the study period (Table 1). The best model also included the individual variables female age (the quadratic effect, Age f^2^), male polygyny status (beta polygyny indicator) and whether the territory occupied by the male was new to him or not (M novelty; Table 1). Among the random effects, the interaction between female (F id) and territory ID accounted for 6.7% of the variation in hatching date, while territory ID itself accounted for 4.3% and the variation in specific river discharge in combination with year accounted for 20.5% (Qspec sd 23, random slope; Table 1) of the variation in hatching date (Table 1). Hatching date advanced (was earlier) with increasing normalized mean winter river discharge (Winter Qnorm mean 17), spring NAO (spring NAO 26), and spring groundwater levels (Spring Groundwater mean 14; Fig. 2). There was a quadratic effect for female age (Age f^2^), with delayed hatching date for females younger than four years old (fifth calender year; Fig. 3) and for females older than that. On the other hand, hatching date was delayed by increasing variation in spring snow cover (Spring SCA sd 6) at the lowest part of each territory’s catchment (Fig. 2), male territory novelty (M novelty) and polygyny (beta polygyny indicator), and altitude (Fig. 4). During the study period, the delay in hatching date decreased from 5.5 to 2.5 days per 100 m increase in altitude (Fig. 4), indicating that the difference in hatching date at high and low altitudes diminished over the study period. The random slope variation in specific river discharge during the previous spring and summer (Qspec sd 23) could not be explained by annual variation or any other predictor variable, although within the interaction, year explained 91% of the variation. The random slope in specific river discharge nevertheless had a significant effect on the timing of breeding (see Figure S3, Additional file 2), and this means that the effect of river discharge the previous spring and summer could be negative in 1 year and positive in another. The 95% confidence interval for the model prediction was ± 10.7 days (SD = 5.5).

**Table 1:** Estimate fixed effects and variance decomposition explaining the variation in breeding time

Variables	Estimate	Std. Error	Variance decomposition
Intercept	136	3.15	–
Spring SCA sd [6]	7.0	2.14	0.4
Spring Groundwater mean [14]	− 0.067	0.02	1.1
Winter Qnorm mean [17]	− 6.6	0.95	10.3
Spring NAO [26]	− 0.78	0.24	1.2
M novely	2.2	0.43	0.8
beta polygyny indicator	6.7	0.73	2.2
Age f	− 5.7	0.67	–
Age f^2^	0.57	0.08	2.8
Altitude	0.055	0.004	–
Year	− 0.10	0.09	–
Altitude × Year	− 0.00082	0.00	29.2

	Std. Deviation	Correlation	–
Year	8.4		–
Qspec sd [23] |Year	0.080	− 0.85	20.5
Territory id	2.5		4.3
F id × Territory id	3.1		6.7
Residual	5.5		–

Estimated fixed effects and variance decomposition of fixed and random effects in a linear mixed effects model explaining the variation in hatching date in the white-throated dipper, where “spring” and “winter” is applied to enhance understanding of the terms SCA (Snow Covered Area), Qnorm (relative river discharge) and NAO (North Atlantic Oscillation). Numbers in brackets in the variable names denote the trigger period, mean and sd denote mean and standard deviation of the variable in the specified trigger period

## Discussion

Timing of breeding advanced 10  days during the study period 1978–2015. Earlier breeding in birds is one of the most reported responses to climate change [14, 38, 42]. Dunn and Winkler [14] argued that variation in population density and habitat quality might influence the interpretation of phenology responses. However, this is unlikely to have been the case in our study. Although the population density of the dipper is predicted to increase in the future because of warmer winters [18], currently there are strong fluctuations but no obvious long-term trend [35]. Previous work in this study system [19, 37] also indicates that high population density results in earlier breeding, which is opposite to the prediction by Dunn and Winkler [14]. The advancement in breeding time reported here may thus be interpreted as a response to climate change.

Environmental variation measured at the territory had a significant influence on the timing of breeding. The level of environmental heterogeneity between territories was immense, as reflected by the variation in the trigger date (the first date when temperature was above 0 °C for five consecutive days), which varied by more than a month on average and in some years almost by 3 months. Thus, individuals even within close range of each other, in this case within 70 km, experienced very different environmental conditions. This supports the findings by Hinks et al. [24], where the timing of oak leaf emergence in the territory accurately predicted breeding phenology. Evidently, there is immense environmental heterogeneity within single populations, and large-scale climate indices or population-level measurements might not reliably capture the level of environmental variation the individuals are exposed to [11, 24].

The environmental conditions prevailing on the territories around the trigger date, but also the winter conditions, affected breeding time. Territory river discharge was particularly influential in determining the breeding time in the dipper, as suggested by D’Amico et al. [12]. Surprisingly, it was not the river discharge immediate before breeding, around the trigger date, but during the winter months prior to the breeding season that had the greatest effect on the breeding time. As the food sources exploited by the dippers are hidden under water, the water levels, velocity and turbidity could influence dipper foraging success during winter [15], which might have implications for when the individual bird is physiologically capable of starting to breed. In general, precipitation in southern Norway has increased (1961–2009; [47] and, similarly, the mean winter temperature in the study area has increased at least by 3 °C (1978–2008; [35], meaning that less precipitation falls as snow. During the study period, there was a tendency towards increased river discharge, which might contribute to the temporal trend in breeding phenology. Most importantly, high discharge indicates open water [26, 40] and thus favourable conditions, because this species is negatively affected by the ice conditions in winter [35]. In the 3–4 weeks prior to the trigger date at each territory, increasing minimum ground water levels and decreasing maximum snow-covered area (SCA) advanced breeding time. This critical time period precedes territory establishment and nest building. The minimum groundwater levels may reflect some aspects of the discharge at a vital time, while the snow conditions may indicate the timing of spring floods. Flooding might be particularly detrimental to the dipper, because the nest is positioned immediately above fast-flowing water and may be flushed away by the current if water levels increase. Flooding may also reduce water visibility and hamper foraging success [8]. Judging from the relative importance of the investigated variables, we conclude that the long-term (months) hydrological conditions specific to a particular territory prior to the breeding season seem surprisingly important compared to the weather conditions prevailing shortly before breeding.

An unexpected effect was the variation in response to discharge variation during the previous spring and summer between years (the random slope). A possible explanation might be that discharge variation might have a very different effect in a year with moderate compared to high mean discharge. However, it may also be that this effect is correlated with an unmeasured climatic driver that vary from year to year.

Dippers breeding at high altitudes were delayed, a finding also reported in other bird species [23, 28, 44, 56]. Altitude was the parameter that contributed the single most to explain the variation in the timing of breeding. As there is a steep altitudinal gradient in this population and temperature drops with 0.65 °C by 100 m increase in altitude, altitude was probably indicating when in spring a territory would become available to prospective breeders. Surprisingly, the altitudinal effect on the timing of breeding was decreasing over the study period, from 6 days to merely 1 day delay in hatching per 100 m increase in altitude. It might be that after mild winters, more territories are accessible prior to the breeding season also at higher elevations, rending the altitudinal gradient less influential. Because of global warming, winters have generally become warmer and this might have led to a more synchronous breeding across altitudes in our study population.

We found a significant interaction between territory and female identity that affected the timing of breeding, i.e., certain combinations of individuals and territories were associated with earlier breeding and others with later breeding. The ability to take advantage of the resources in a given territory may differ between individuals and be reflected by the timing of breeding. In some species, immigrants and locally recruited birds may differ both in laying time and prey choice, and this may reflect genetic differences, differences in quality, or the early learning environment [50]. Interestingly, males that established themselves in unknown territories as well as first-time breeding males, bred on average 2 days later than males that established themselves in familiar territories where they had previous breeding experience. Thus, not only female experience but also male familiarity with a territory may influence the time of breeding. Female site familiarity has previously been shown to be important [45], but it has only recently been shown that male experience influence breeding time [57]. Foraging and prey choice may however improve equally with experience in the two sexes [50]. Depending on their local experiences, males might differ in eagerness and contribution to nest building, thus affecting the time for initiation of breeding. This finding highlights the importance of male territory familiarity and thus proving that breeding time is not solely a female trait.

We also found that breeding time depended on female age, with four-year olds breeding earlier than both younger and older females. Reproductive performance and associated traits such as the timing of breeding usually improve with age and then level off [16]. However, age-specific reproductive output is more commonly reported than age-specific differences in timing of breeding (but see [5, 20, 30, 32, 53, 59]. Supposedly, the same mechanisms governing age-specific reproductive output, such as selective disappearance of poor breeders and individual improvement with increasing age and experience [20, 32, 41], might also explain the observed pattern in breeding phenology.

Polygyny is well documented in the dipper, but the frequency varies both between populations and years [17, 31, 58]. We found that breeding time did not differ between females mated to monogamous males and primary females mated to polygynous males, but that the onset of breeding of females that settled with an already mated male was delayed. In this species, late breeding reduces reproductive success [37] which might be a significant cost of polygyny.

## Conclusions

In conclusion, we have shown how hydrological dynamics and other prominent local characteristics of the environment at the territory scale influence the timing of breeding in the dipper. Particularly, the river discharge and the altitudinal effect were of great importance to this riverine specialist bird. From a management perspective, it should be emphasized that the dipper, often used as an indicator species, is particularly sensitive to changes in river discharge and the snow conditions during the entire winter period as well as in early spring. This also applies to rivers and streams outside the breeding areas, because the dipper is a short-distance partial migrant. This study is by nature an explorative approach, where the results provide the first step in seeking out biologically relevant hydrological and other environmental drivers of the timing of breeding. Further work is required to understand how discharge is affecting riverine birds in the long-term, particularly in the immediate proximity to hydropower developments.

## Methods

### Study species and study system

The dipper is common across the Palearctic mountainous regions and it breeds in close proximity to rapidly flowing parts of rivers and streams. Territories are established in early spring and both sexes build the nest and feed the young, except in instances of polygyny where the male contribution is lower. The clutch of 3–6 eggs are incubated by the female for a period of 17 days and the young remain in the nest for approximately 20 days before fledging [19, 37, 52]. The study population is partially migratory, where part of the population undertakes migration to southern Sweden, Denmark, and northern Germany and Poland [4].

The study system is situated in river Lyngdalselva and its tributaries in southernmost Norway (58° 08′–58° 40′ N, 6° 56′–7° 20′ E). The system is subject to a strong altitudinal gradient, reaching almost from the river mouth in Lyngdalsfjorden to 60 km inland and 700 m above sea level. The population has been monitored according to a standardised methodology since 1978 [35]. The population size has fluctuated between 20 and 117 pairs (defined as the number of breeding females). Almost all (94%) breeding birds have been individually identified. Birds are caught in mist nets at first encounter and ringed with a metal ring and an individual colour code comprising two plastic rings. Individual colour codes enable later recognition of individual identity without having to recapture the bird. Arrival date was registered as first encounter of each bird each year, by ring reading or capture at first encounter. In some instances, arrival date was not registered until well into the breeding cycle. The breeding outcome of almost all nests is known and nearly all young are ringed. For more details on the study system, see Nilsson et al. [35]. Dippers prefer nest sites where the opening of the nest is situated over fast-flowing water [52]. Because nest sites are limited and spatially segregated, there is a limited number of individual territories containing one or more nest sites, namely 158, as recorded within the whole river system during the entire study period.

The timing of breeding is estimated as the hatching date of the first clutch of a female in a year (see [36]. In short, hatching date is based on the growth trajectory of the largest young in the brood, i.e. the individual that is closest to the maximum physiological growth rate. This implies that the phenology of nests that did not produce young, or produced young that were not measured, could not be estimated by this method. All-together, a total of 1184 breeding events with estimated breeding time during the study period are analysed here. A total of 1271 breeding events with no estimated breeding time were excluded, and another 209 breeding events with breeding time were excluded due to inaccuracies in the ringing data or absence of hydrology data.

### Hydrology data

Hydrological data are normally restricted to gauged sites, which often are inconveniently located with respect to the biological study systems of interest. In Lyngdalselva, only two sites are gauged. However, the rainfall-runoff model (the Distance Distribution Dynamics (DDD) model, [48]) has produced predictions of river discharge in ungauged basins in the Lygne basin at each unique dipper breeding territory (except for 13 out of 158 territories whose catchments were too small). The model for Lyngdalselva is extremely good when comparing model predictions and gauged sites; the Kling Gupta Efficiency criterion (KGE; [21, 27]) for both gauged sites in Lyngdalselva is 0.94. Thiemig et al. [51] regard values KGE 0.5–0.75 as intermediate, and 0.75–0.9 as good; a KGE of 0.94 for Lyngdalselva is thus extraordinarily good. Several of the model parameters of DDD are estimated from digitized maps of terrain and river network. In addition, regressed relations between model parameters and catchment characteristics, like fractions of forest and bare rock, wetland, and so on are determined by calibrating the model against observed runoff from several gauged catchments in Norway. Daily river discharge data in the Lygne basin have therefore been estimated for the whole study period 1978–2015. The data input for the rainfall-runoff-model, precipitation (P; Table 1) and temperature (T; Table 2), stems from the interpolated meteorological grid, and contains also environmental variables specific to each territory. In addition, snow covered area (SCA; Table 2), snow water equivalent (SWE; Table 2; [49] and groundwater levels (Groundwater; Table 2) are predicted by the DDD model. All are variables that may prove to be significant biological predictors of the timing of breeding in the dipper, reflecting microclimatic or other important environmental variation at each territory (for a full list, see Table 2; for further details on the hydrological variables, see [48].

**Table 2:** Predictor variables and their explanation used in analysing variation in breeding time

Variable type	Predictor	Explanation
Territory–indicating microclimate	Altitude	Altitude of most frequented nest site at the territory
	Distance^a^	Distance from the river outlet to the most frequented nest site at the territory
	Qmean^a^	Absolute mean annual discharge
Catchment–indicating microclimate	Area	Size of catchment area (m^2^)
	Altitude median	Median elevation of catchment (m)
	Altitude diff	Altitudinal range of catchment (m)
	Bog fraction	Fraction of bogs in catchment, which contribute to flow when saturated
	Bog max	Maximum of distance distribution for bogs (m)
	Bog mean	Mean of distances (m) between grid-points in the catchment classified as bogs to the nearest river reach, indicating the flow dynamics from saturated bogs
	Bog fraction^b^	Areal fraction of grid cells with zero distance to the river network for bogs
	S mean	Mean of distances (m) between grid-points in the catchment classified as soils to the nearest river reach, indicating the flow dynamics from soils
	Soil fraction	Areal fraction of grid cells with zero distance to the river network for soils
	L mean	Mean of the distances (m) between points in the river network, a measure of river network complexity
	L sd	Standard deviation of distances between points in the river network to the outlet, a measure of river network complexity
	L max	Maximum of distance distribution of the river network, a measure of river network complexity
	Evotranspiration	Degree-day factor for evotranspiration (mm/day/ °C)
Trigger	Trigger day	Trigger day index for first five-day period above 0 °C
	Qspec*	Specific discharge (l/s/km^2^, discharge per area unit)
	Qnorm*	Normalized discharge (discharge/annual mean discharge)
	T*	Temperature (°C)
	P*	Precipitation (mm)
	Groundwater*	Dynamic groundwater storage (mm). The part of groundwater storage that fluctuates with runoff indicating hydrological drivers
	SCA*	Fraction of the lowest 10% of the catchment that is snow covered
	SWE*	Snow water equivalent at the lowest 10% of the catchment (mm)
	Ql10*	Indicating whether discharge exceeded 10 l/s or not (indicating extreme drought) in period
	Ql100*	Indicating whether discharge exceeded 100 l/s or not (indicating drought) in period
Global climate	NAO*^b^	Daily North Atlantic Oscillation Index
	NAOcurr	North Atlantic Oscillation Index current year
	NAOprev	North Atlantic Oscillation Index previous year
Individual	Age f^a^	Female age indicating individual experience and possible age restrictions
	Age m^a^	Male age indicating individual experience and possible age restrictions
	Polyandry	Polyandry status: monogamous or polyandrous brood
	Polygyny all	Polygyny status: monogamous, alpha, beta, gamma, successive alpha, and successive beta brood
	Polyandry history	0/1 indicator for whether the female has been polyandrous, 1 = yes
	Polygyny history	0/1 indicator for whether the male has been polygynous, 1 = yes
	polygyny indicator	0/1 indicator for whether the male was detectably polygynous for this brood, 1 = yes
	beta polygyny indicator	0/1 indicator for whether the male was detectably polygynous and this wasn’t the alpha brood, 1 = yes
	polyandry indicator	0/1 indicator for whether the female was detectably polyandrous for this brood, 1 = yes
	male first	0/1 indicator for whether this was the first hatching for this male, 1 = yes
	female first	0/1 indicator for whether this was the first hatching for this female, 1 = yes
	M novelty	Male territory novelty, 1 = first time for this male in this territory
	F novelty	Female territory novelty, 1 = first time for this female in this territory
Other	lintime	Temporal time trend, indicating possible global climatic change
Random effects	Year^a^	Annual variation not explained by climatic variables
	M id	Male identity (ring number) indicating individual variation
	F id	Female identity (ring number) indicating individual variation
	Territory id	Territory identity indicating spatial variation not explained by territory and catchment variables

A list of all predictor variables and their biological explanation used in analysing the variation in the timing of breeding in the white-throated dipper population in Lyngdalselva 1978–2015

*Mean, min, max and sd were estimated for all trigger periods

^a^The quadratic effect was also included

^b^From the National Weather service, Climate Prediction Center, http://www.cpc.ncep.noaa.gov/products/precip/CWlink/pna/nao.shtml

In the study area, territory river discharge is of vastly different magnitudes. For example, the last dipper territory in the main river before the outlet into the fjord has a very large catchment area and thus discharge compared to the territories located in small brooks in the mountains (mean discharge varied between territories, from 0.008 m^3^/s to 32 m^3^/s). To allow comparison among territories, river discharge was standardized with two competing methods. First, river discharge was standardized as specific discharge, which is discharge per catchment area, measured in l/s/km^2^, at each breeding territory (Qspec; Table 2). Second, it was standardized as the relative river discharge (defined as discharge divided by the territory mean discharge for the study period; Qnorm; Table 2).

Because access to open water is of vital importance for the dippers’ foraging and thus breeding opportunities in seasonal environments, we defined an annual trigger date based on the daily temperature at each individual territory (Additional file 1). Hence, the trigger date was used for solving the problem of which environmental variable at which time period was of importance for the timing of breeding, with a biological basis. The hydrological simulations produced daily measurements, for 38 full years, of many potentially important hydrological and other environmental variables for timing of breeding in the dipper. The common procedure in dealing with temporal data is to use a sliding window approach [2], which is far from ideal when the period length is unknown and when it likewise is unknown whether the period relates to a date or a biological trigger. By using the response variable, which in studies of phenology is temporal, to find the time period of the exploratory variable with the best fit, the final analysis predicting the response with the best-fit exploratory variable is using the future to predict the past, and thus not a statistically sound procedure. Instead, we solve the problem by allowing the main analysis of the response to find the time period that actually explains the response. The statistics is expanded upon in the Statistics section, but we simply let a multivariate framework select the time period of the explanatory variable(s) that explains the variation in the response. Time periods had a biological basis, built on the “trigger date”; thus, at each territory, the annual trigger date was defined as the first date when the daily air temperature had exceeded 0 °C for five consecutive days (Additional file 1). Such a duration of time will speed up the melting of snow and ice. We then defined a number of different time periods, defined as trigger periods, with different starting dates and of various duration related to the trigger date (see Table S1, Additional file 1). This was done in order to catch weather variation at and around the critical moments when egg laying first becomes feasible (Additional file 1). In addition, some of our possible periods are statically defined, using fixed start and end dates. For each trigger period in each year and territory, we estimated the mean, maximum, minimum and standard deviation. For river discharge, this was calculated for specific discharge as well as for relative discharge. In addition, we introduced two indicator variables for drought, using the original discharge threshold at 0.01 m^3^/s and 0.1 m^3^/s. Lastly, we included the daily North Atlantic Oscillation index (NAO) to account for fluctuations in the global climate [25], that might not have been captured by the variation in the hydrological simulations.

### Statistics

In observational studies, explanatory variables are often correlated. It is thus not unusual that a variable seems to have an influence on the response (in our case the hatching date), simply by being correlated to another variable that influences the response. Multiple regression is therefore important, because the effect of a variable on the response is tested while controlling for all other significant explanatory variables. Thus, in order to avoid spurious correlations we need all the potential explanatory variables that are available and can reasonably affect the response, but due to the many variables we also require a conservative model selection strategy, to reduce the risk of false positives. We used an information criterion for model selection to compare non-nested models in a step-wise search where addition, removal and replacement of covariates were tried. In particular, we chose the Bayesian Information Criterion (BIC) since it is conservative and converges to the true model that is in the set of models considered and if not, to the model closest in Kullback–Leibler distance [9].

Because dippers breed very early in spring in response to warm temperatures [37] and are dependent on submerged invertebrate food, river discharge are among the most likely cues for timing of breeding. We explored some of the core explanatory variables of particular interest for hatching date, namely the trigger date and the winter river discharge, by investigating the univariate regression of each of these variables in turn. We then modelled hatching date with multiple effects using linear mixed-effects models to account for random effects, such as male and female ID, territory ID and year. All analyses were conducted in the programming environment R (version 3.2.5) with add-on packages ‘lme4’ for linear mixed-effects models [6]. Possible predictors included the hydrological variables and other climatic variables such as precipitation, temperature and NAO using the trigger periods (Table 2). Location-specific variables from the rainfall-runoff model were also added as possible predictors, as well as altitude and random location intercepts. We investigated temporal trends in breeding time both by using numeric year as a possible linear predictor and by using categorical year as a random intercept or a random slope for climatic variables. Last, we included the individual characteristics of the breeding birds, namely male and female age, territory novelty (M and F novelty; Table 2) and polygamy status (Polyandry, Polygyny all, etc.; Table 2). For a full list of predictor variables used in modelling the timing of hatching, see Table 2. A discussion of model uncertainty can be found in Additional file 3.

Variance decomposition on the resulting model deemed best according to BIC was performed by looking at the variance contributions for the variable in the model by itself, plus the residual variance. Thus, for fixed factors, we used the variance of the regression coefficient multiplied by the variables’ values in the dataset. This is the same as the squared standardized regression coefficients (see for instance [1], an often-used measure of the relative contribution of variables. For random factors, the variance contribution is listed in the model summary. These variances would sum up to the overall variance of the response if all effects were independent of each other. This was not always the case, which must be kept in mind when interpreting such decompositions. In the case where many quite similar variables were included (such as many different discharge contributions), we used the variance contributions for the set of such variables, which for fixed effects meant the variance of the sum of coefficients multiplied by the variables. This should at least alleviate the problem of variance decomposition due to covariation in the explanatory variables. In addition, we compared the variation in hatching date in the first years of the study period (1978–1996) with the last years (1997–2015) using the F-test.

## Supplementary information

**Additional file 1.** Trigger date and periods. The defined trigger periods, relating trigger date or absolute dates, used on the runoff and catchment predictor variables when modelling timing of breeding in the white-throated dipper in Lyngdalselva 1978–2015

**Additional file 2.** Variation in specific discharge affects timing of breeding. The effect of the standard variation in specific discharge during the period Apr–Aug the preceeding year on the timing of breeding (hatching day-of-year), where each year is denoted in a different colour, in the white-throated dipper in Lyngdalselva 1978–2015

**Additional file 3.** Model uncertainty. Alternative models with the defined trigger periods, relating trigger date or absolute dates, used on the runoff and catchment predictor variables when modelling timing of breeding in the white-throated dipper in Lyngdalselva 1978–2015

## Data Availability

The datasets analysed during the current study are not publicly available, due to it being a unique long-term collection of individual data on a breeding dipper population, which has cost an enormous effort in the field 1973–present, but are available from the corresponding author on reasonable request. The complete dipper data are stored at the Norwegian University of Life Sciences.

## Abbreviations

NAO: North Atlantic Oscillation
SD: Standard Deviation
Qnorm: Normalized river discharge, which is discharge divided by territory mean discharge for the study period
Qspec: Specific river discharge, discharge per catchment area l/s/km^2^
Groundwater: Ground water levels
SCA: Snow Covered Area
Age f: Female age
Age m: Male age
Age f^2^: Quadratic female age
beta polygyny indicator: Male polygyny
F novelty: Whether a territory was new to a female
M novelty: Whether a territory was new to a male
F id: Female identity
M id: Male identity
territory ID: Territory identity
trigger date: The first date when temperature was above 0 degrees Celsius for five consecutive days
trigger periods: Time periods with different starting dates and of various duration related to the trigger date: in addition to some periods with fixed start and end date
DDD: Distance Distribution Dynamics model
KGE: Kling Gupta Efficiency criterion
P: Precipitation
T: Temperature
SWE: Snow water equivalent
BIC: Bayesian Information Criterion
