# Supplementary material for: Hydrology influences breeding time in the white-throated dipper
Source: BMC Ecol. 2020 Dec 17;20:70. doi: 10.1186/s12898-020-00338-y (PMC7745505; doi:10.1186/s12898-020-00338-y)
Supplement: Supplementary file 1 — Additional file 1. Trigger date and periods. The defined trigger periods, relating trigger date or absolute dates, used on the runoff and catchment predictor variables when modelling timing of breeding in the white-throated dipper in Lyngdalselva 1978–2015 [file 12898_2020_338_MOESM1_ESM.docx]

Additional file 1. Trigger date and periods

Anna L. K. Nilsson, Thomas Skaugen, Trond Reitan, Jan Henning L’Abée-Lund, Marlène Gamelon, Kurt Jerstad, Ole Wiggo Røstad, Tore Slagsvold, Nils C. Stenseth, L. Asbjørn Vøllestad & Bjørn Walseng

Corresponding author: [anna.nilsson@ibv.uio.no](mailto:anna.nilsson@ibv.uio.no), tel: +47 22859049, fax: 22854001

Trigger date and periods

When explaining a biological event with an abiotic variable that is changing over time, one of the challenges is to find the time period of biological interest. Access to open water is of the utmost importance for the dipper. We therefore defined annual trigger dates as the first date when the daily temperature exceeded 0°C for five consecutive days at each territory. Our aim was that the trigger date should be timed before the birds started laying and thereby reflect the environmental conditions birds might use as cues for the timing of breeding. The trigger date was therefore a compromise between the temperature with the best correlation with hatching date and the fact that the trigger date could not “exceed” hatching date – three weeks, the approximate time required for laying and incubation (Tyler and Ormerod 1994). We then defined a number of different time periods, which we termed trigger periods, with different starting dates and of variable duration related to the trigger date. The trigger periods were selected as to encompass early, mid and late periods of short and long duration. For each trigger period, we estimated the mean, maximum, minimum and standard deviation of runoff for each year and territory. This was calculated for area specific as well as normalised runoff.

Table A1. The defined trigger periods, relating trigger date or absolute dates, used on the runoff and catchment predictor variables when modelling timing of breeding in the white-throated dipper in Lyngdalselva 1978-2015

| Trigger period | Start | End | Index | Note |
| --- | --- | --- | --- | --- |
| 1 | -30 | -15 | 0 |  |
| 2 | -20 | -5 | 0 |  |
| 3 | -10 | -1 | 0 |  |
| 4 | -5 | 4 | 0 |  |
| 5 | -40 | -15 | 0 |  |
| 6 | -30 | -1 | 0 |  |
| 7 | -15 | 10 | 0 |  |
| 8 | -40 | -25 | 0 |  |
| 9 | -50 | -35 | 0 |  |
| 10 | -40 | -1 | 0 |  |
| 11 | 0 | 9 | 0 |  |
| 12 | -15 | 0 | 0 |  |
| 13 | -15 | -6 | 0 |  |
| 14 | -20 | 0 | 0 |  |
| 15 | -20 | -10 | 0 |  |
| 16 | 0 | 59 | 1 | Jan-Feb |
| 17 | 334 | 59 | 1 | Dec-Feb |
| 18 | -365 | -1 | 0 | Previous year |
| 19 | -182 | -1 | 0 | Previous 6 months |
| 20 | 243 | 333 | 1 | Previous Sep-Nov |
| 21 | 151 | 242 | 1 | Previous June-Aug |
| 22 | 91 | 151 | 1 | Previous Apr-June |
| 23 | 91 | 242 | 1 | Previous Apr-Aug |
| 24 | 151 | 333 | 1 | Previous June-Nov |
| 25 | 5 | 15 | 0 |  |
| 26 | 10 | 21 | 0 |  |
| Index: 0=relative trigger, 1=absolute dates | | | | |
