# Supplementary material for: Hydrology influences breeding time in the white-throated dipper
Source: BMC Ecol. 2020 Dec 17;20:70. doi: 10.1186/s12898-020-00338-y (PMC7745505; doi:10.1186/s12898-020-00338-y)
Supplement: Supplementary file 2 — Additional file 2. Variation in specific discharge affects timing of breeding. The effect of the standard variation in specific discharge during the period Apr–Aug the preceeding year on the timing of breeding (hatching day-of-year), where each year is denoted in a different colour, in the white-throated dipper in Lyngdalselva 1978–2015 [file 12898_2020_338_MOESM2_ESM.docx]

Additional file 2. The effect of the standard variation in specific discharge during the period Apr-Aug the preceeding year on the timing of breeding

Anna L. K. Nilsson, Thomas Skaugen, Trond Reitan, Jan Henning L’Abée-Lund, Marlène Gamelon, Kurt Jerstad, Ole Wiggo Røstad, Tore Slagsvold, Nils C. Stenseth, L. Asbjørn Vøllestad & Bjørn Walseng

Corresponding author: [anna.nilsson@ibv.uio.no](mailto:anna.nilsson@ibv.uio.no), tel: +47 22859049, fax: 22854001


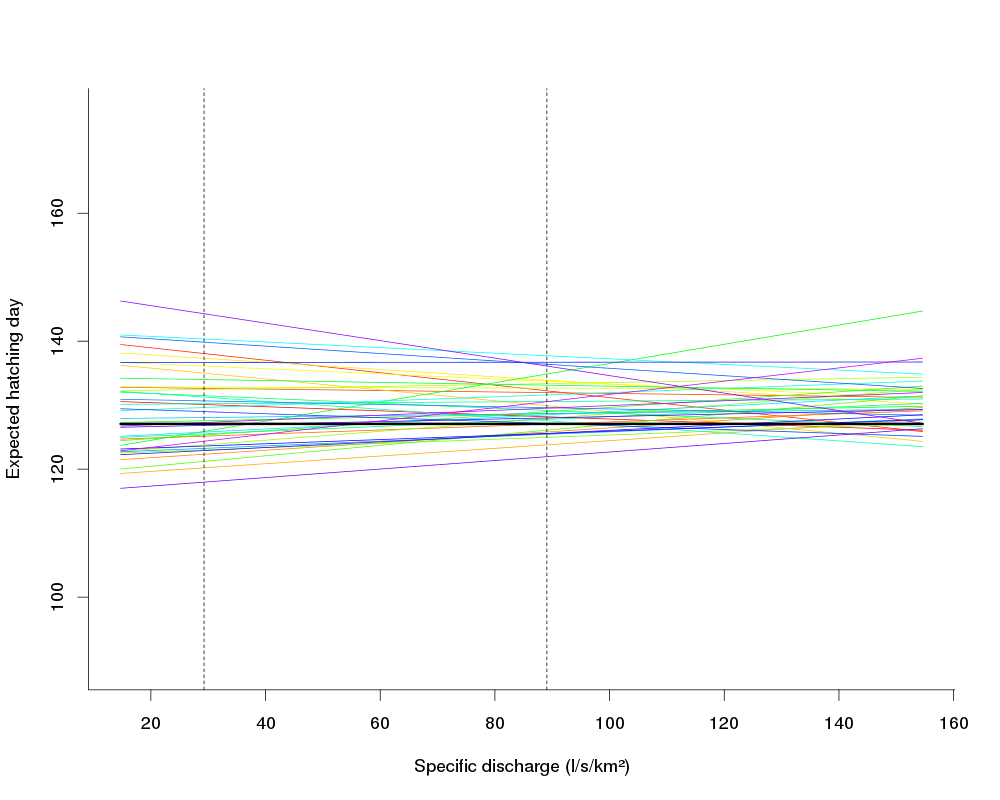


Figure S3. The effect of the standard variation in specific discharge during the period Apr-Aug the preceeding year on the timing of breeding (hatching day-of-year), where each year is denoted in a different colour, in the white-throated dipper in Lyngdalselva 1978-2015.
