# Supplementary material for: Hydrology influences breeding time in the white-throated dipper
Source: BMC Ecol. 2020 Dec 17;20:70. doi: 10.1186/s12898-020-00338-y (PMC7745505; doi:10.1186/s12898-020-00338-y)
Supplement: Supplementary file 3 — Additional file 3. Model uncertainty. Alternative models with the defined trigger periods, relating trigger date or absolute dates, used on the runoff and catchment predictor variables when modelling timing of breeding in the white-throated dipper in Lyngdalselva 1978–2015 [file 12898_2020_338_MOESM3_ESM.docx]

Additional file 3. Model uncertainty

Anna L. K. Nilsson, Thomas Skaugen, Trond Reitan, Jan Henning L’Abée-Lund, Marlène Gamelon, Kurt Jerstad, Ole Wiggo Røstad, Tore Slagsvold, Nils C. Stenseth, L. Asbjørn Vøllestad & Bjørn Walseng

Corresponding author: [anna.nilsson@ibv.uio.no](mailto:anna.nilsson@ibv.uio.no), tel: +47 22859049, fax: 22854001

Model uncertainty

The uncertainty of the outcome of an information criterion based model selection can be expressed by reporting the models that are closest to the best information criterion. A relative weight can be calculated for these models, which for the BIC can be viewed as an approximation to the Bayesian model probability (see Schwartz 1978, where a different scaling than the modern definition for the BIC value was used). Here we report 10 alternative models (including the model presented in the main text) that had the lowest BIC values of the models that were traversed in the step-wise model search.

The alternative models were very similar to the top model (model 1) reported in the main text (Table A2). All of the predictor variables included in the top model are included in the alternative models, except Qnorm.mean.17 and NAO.26. However, while Qnorm.mean.17 can be said to denote the largest difference between the top ten models, this uncertainty consists simply of whether to use relative mean winter discharge (Qnorm.mean.17) or specific mean winter discharge (Qspec.mean.17), for the same trigger period. ). Similarly, NAO.26 is replaced by P.mean.01 in two models, indicating that NAO might reflect precipitation patterns. In addition to mean winter discharge, the minimum winter discharge for the same time period was included in two of the ten top models (model 8 and 9). Moreover, six of the alternative models also include SCA.max.01 (model 2 and 3), Groundwater.min.17 (model 10), distance from the coast (model 5 and 6) and trigger day (model 7). The possible additional variables SCA.max.01, minimum winter discharge and groundwater.min.17 can be said to modify the effect of climate variables that are already there, by either introducing an extra period (SCA.max.01, groundwater.min.17) or by introducing an additional statistics for an already included period (minimum winter discharge). All in all, the structure of the top model is retained, just with a possibility of replacement for two variables (Qnorm.mean.17->Qspec.mean.17 and NAO.26->P.mean.01) and addition of some extra variables.

Table S2. Alternative models with the defined trigger periods, relating trigger date or absolute dates, used on the runoff and catchment predictor variables when modelling timing of breeding in the white-throated dipper in Lyngdalselva 1978-2015

| Predictor variables | Model 1 | Model 2 | Model 3 | Model 4 | Model 5 | Model 6 | Model 7 | Model 8 | Model 9 | Model 10 |
| --- | --- | --- | --- | --- | --- | --- | --- | --- | --- | --- |
| SCA.max.01 |  | x | x |  |  |  |  |  |  |  |
| SCA.max.06 | x | x | x | x | X | x | x | x | x | x |
| Groundwater.min.14 | x | x | x | x | X | x | x | x | x | x |
| Groundwater.min.17 |  |  |  |  |  |  |  |  |  | x |
| Qnorm.mean.17 | x | x |  |  | X |  |  | x | x |  |
| Qspec.mean.17 |  |  | x | x |  | x | x |  |  | x |
| Qnorm.min.17 |  |  |  |  |  |  |  |  | x |  |
| Qspec.min.17 |  |  |  |  |  |  |  | x |  |  |
| P.mean.01 |  |  |  |  |  |  | x |  |  | x |
| NAO.26 | x | x | x | x | X | x |  | x | x |  |
| M novelty | x | x | x | x | X | x | x | x | x | x |
| beta polygyny indicator | x | x | x | x | X | x | x | x | x | x |
| Age f^2^ | x | x | x | x | X | x | x | x | x | x |
| Distance |  |  |  |  | X | x |  |  |  |  |
| Trigger day |  |  |  |  |  |  | x |  |  |  |
| Territory id | x | x | x | x | X | x | x | x | x | x |
| F id:Territory id | x | x | x | x | x | x | x | x | x | x |
| Qspec sd (23)\|Year | x | x | x | x | x | x | x | x | x | x |
| Altitude x Year | x | x | x | x | x | x | x | x | x | x |
| BIC | 7924.23 | 7924.24 | 7924.50 | 7924.53 | 7924.54 | 7924.63 | 7924.66 | 7924.89 | 7924.92 | 7924.94 |
| BIC-weights (%) | 12 | 11.96 | 10.45 | 10.31 | 10.27 | 9.83 | 9.65 | 8.62 | 8.49 | 8.43 |

References

Schwarz, Gideon E. (1978), "Estimating the dimension of a model", *Annals of Statistics,* 6 (2): 461–464.
